# Development of the Self-efficacy for Social Participation scale (SOSA) for community-dwelling older adults

**DOI:** 10.1186/s12889-023-16774-6

**Published:** 2023-11-20

**Authors:** Nanami Oe, Etsuko Tadaka

**Affiliations:** 1 Department of Community and Public Health Nursing, Graduate School of Health Sciences, Hokkaido University, N12-W5, Kita-Ku, Sapporo, Hokkaido 060-0812 Japan; 2 Department of Community and Public Health Nursing, Faculty of Health Sciences, Hokkaido University, N12-W5, Kita-Ku, Sapporo, Hokkaido 060-0812 Japan

**Keywords:** Scale development, Self-efficacy, Social participation, Community, Older adults, Public health

## Abstract

**Background:**

Social participation is important for the health of older adults and super-aging societies. However, relatively few independent older adults in advanced countries actually participate in society, even though many of them have the capacity to do so. One possible reason for this could be a lack of self-efficacy for social participation. However, few scales have been developed to measure self-efficacy for social participation among community-dwelling independent older adults. Therefore, we developed the “Self-efficacy for Social Participation” scale (SOSA) to assess the self-efficacy of community-dwelling independent older adults, and examined the scale’s reliability and validity.

**Methods:**

We distributed a self-administered mail survey to approximately 5,000 randomly selected independent older adults throughout Japan. The construct validity of the SOSA was determined using exploratory and confirmatory factor analyses. Criterion-related validity was assessed using the General Self-Efficacy Scale (GSES) and according to subjective health status.

**Results:**

In total, 1,336 older adults responded to the survey. Exploratory and confirmatory factor analyses identified 12 items distributed among four factors: instrumental self-efficacy, managerial self-efficacy, interpersonal self-efficacy and cultural self-efficacy. The final model had a Cronbach’s alpha of 0.90, goodness-of-fit index of 0.948, adjusted goodness-of-fit index of 0.915, comparative fit index of 0.952, and root mean square error of approximation of 0.078. Significant correlations existed between the SOSA score and GSES (*r* = 0.550, *p* < 0.01) and subjective health status (*r* = 0.384, *p* < 0.01) scores.

**Conclusions:**

The SOSA showed sufficient reliability and validity to assess self-efficacy for social participation among older adults. This scale could aid efforts to improve the physical and mental health, and longevity, of older adults through increased behavioralizing social participation.

**Supplementary Information:**

The online version contains supplementary material available at 10.1186/s12889-023-16774-6.

## Background

According to a report from the World Health Organization, the average life expectancy in Japan was 81.5 years for men and 86.9 years for women in 2019, which represent the longest lifespans in the world. However, the healthy life expectancies are shorter, at 72.6 years for men and 75.5 years for women [1]. Healthy life expectancy is the average length of time during which there are no restrictions on daily life [2]. Over the past 15 years, the average life and healthy life expectancies have increased for both men and women [3]. However, the gap between the average life expectancy and healthy life expectancy is large, at 8.9 years for men and 11.4 years for women; in other words, people live with some restrictions on their daily lives for about 10 years. Extending healthy life expectancy is considered an important issue in advanced countries as we approach the era of 100-year lifespans.

Social participation plays a crucial role in the health and well-being of older adults. Levasseur et al. define social participation as “a person’s involvement in activities that provide interaction with others in society or the community” [4]. Research has shown that socially active older adults, such as those involved in neighborhood associations, have a significantly lower risk of prefrailty [5]. Additionally, engaging in exercise-based social participation has been linked to recovery from frailty [6]. Furthermore, social participation has been associated with the maintenance of daily activities, including instrumental activities of daily living, as well as cognitive function [7, 8]. Given the positive impact of social participation on the physical and mental health of older adults, it is imperative to promote social engagement in this population to prevent the need for long-term care and enhance healthy life expectancy.

However, despite the potential benefits, a substantial number of older adults do not participate in social activities. In a study of older adults in Japan, 26.4% of 65–74-year-olds participated in “local-government organizations” (resident and neighborhood associations), while 4.2% participated in “community development and community safety”, 18.2% in “volunteering and social service through hobbies and sports”, 1.8% in “traditional performing arts and craft techniques”, 1.9% in “life support and child-rearing support”, and 3.7% in “other” activities. However, 59.8% of respondents did not engage in any particular activity [9]. Furthermore, a study of 51,302 physically and cognitively independent persons aged ≥ 65 years living in 12 municipalities in Japan found that 54.1% had never belonged to a hobby activities group, 72.8% had never belonged to a sports group or club, and 82.2% had never belonged to a volunteer group [10]. These findings suggest that a significant proportion of Japanese older adults, estimated to be around 60%–80%, do not actively participate in any social activities. Similar trends have been observed in studies conducted in the United States and Europe, indicating that low social participation is not limited to Japan but is instead an issue among older individuals worldwide [11, 12].

To extend healthy life expectancy, it is crucial to address the factors contributing to a lack of social participation among independent older adults. One potential reason for this phenomenon is a lack of self-efficacy. Social participation requires older individuals to possess various self-efficacies in different domains. Moreover, access to public transportation and related information is crucial to facilitate social participation [13–15]. Positive perceptions of the user-friendliness of the walking environment have also been associated with higher levels of social participation [16]. In addition, from an interpersonal perspective, individuals who are willing to communicate with their neighbors or friends are more likely to engage in social activities [17]. Moreover, a desire for social connectedness and enjoyment of group activities play significant roles in motivating older adults to participate therein [18]. Being personally invited, and feeling valued and acknowledged, further promotes social participation [15]. At the managerial level, health concerns and lack of confidence in physical strength were commonly cited by older adults as reasons for not engaging in social activities [19]. Flexibility in activity scheduling, including volunteering, has been identified as important for promoting social participation [15]. From a cultural perspective, access to information about available activities and services is crucial for encouraging older adults to participate in society [15]. Additionally, factors such as motivation to learn, personal interests, and the desire for personal growth can influence the willingness to engage in new interactions [20]. Lastly, older adults with greater social orientation tend to be more involved in social activities [21]. Considering these findings, it is evident that social participation among older adults requires the development of self-efficacy in the instrumental, interpersonal, managerial, and cultural domains.

According to Bandura, self-efficacy refers to recognition of the possibility that an individual can effectively perform the necessary actions in a certain situation [22]. Bandura also distinguished two types of self-efficacy: general and specific self-efficacy. General self-efficacy relates to an individual's belief in their overall ability to handle various challenging situations and achieve desired outcomes. It refers to a broad and generalized sense of confidence in one’s own capabilities to succeed in different life domains. People with high levels of general self-efficacy tend to have confidence in their problem-solving skills, resilience, and adaptability across a wide range of situations. They view challenges as opportunities for growth and are more likely to take on new tasks and persist in the face of obstacles. Specific self-efficacy refers to an individual's belief in their ability to perform particular tasks or activities successfully within a specific domain or context. This relates to the confidence an individual has in their capacity to accomplish specific goals, acquire specific skills, or execute specific behaviors. Specific self-efficacy beliefs are context-dependent and can vary across life domains, such as academic tasks, athletic performance, social interactions, and specific health behaviors.

In summary, general self-efficacy reflects an individual’s overall belief in their own competence and adaptability across various life domains, while specific self-efficacy pertains to the belief in one’s capability to perform well in specific, context-dependent tasks or activities. Both types of self-efficacy influence individuals’ motivation, choices, and behaviors and are important considerations when seeking to understand how people approach and navigate various challenges and opportunities in life. However, specific self-efficacy is thought to play a particularly important role in social participation among older adults, enabling them to lead healthy and fulfilling lives by enhancing their confidence in specific social participation-related skills and abilities, as well as their ability to cope with health challenges and adapt to changes in the environment. However, to date, studies have been limited to general self-efficacy [23–26], or to specific self-efficacy in the context of volunteering [27, 28] or social participation by people with mental illness [29]. Specific self-efficacy has not yet been fully explored in the context of older adults’ social participation.

Developing a self-efficacy scale for social participation among older adults will be crucial for planning, implementing, and evaluating public health policies aimed at promoting healthy longevity among the increasingly large worldwide population of older adults. First, self-efficacy plays a vital role in motivating older adults to participate socially. By developing a self-efficacy scale for social participation, policymakers and implementers can assess the levels of self-efficacy among older adults and plan appropriate support and interventions. For instance, programs and resources could be provided for older adults with low self-efficacy in social participation to support healthy longevity. Second, a self-efficacy scale for social participation would be a valuable tool for quantitative assessment of the effectiveness of public health policies and interventions, including those aimed at improving older adults’ self-efficacy in social participation. On the basis of evaluation results, strategic decisions could then be made to maximize the effectiveness of public health policies, again including those aimed at improving social participation and healthy longevity among older adults. Third, developing a self-efficacy scale for social participation would facilitate knowledge sharing related to the effects of policy on social participation among older adults, across regions and countries, by allowing the collection of standardized data. In this manner, policymakers and researchers would be able learn from each other’s best practices. Improving policies on the basis of information sharing and comparisons at the international level will be highly beneficial in promoting social participation and healthy longevity among older adults.

Therefore, we developed the Self-efficacy for Social Participation scale (SOSA) to assess the self-efficacy of community-dwelling older adults for social activities, and examined its reliability and validity in this study. Herein, self-efficacy for social participation is operationally defined as “the perceived ability to participate in activities shared in time and space with others”.

## Methods

### Phase 1: instrument development

First, to generate a pool of potential items for the SOSA, we conducted a literature review using PubMed and the following search terms: older people OR older adults OR community-dwelling older adults OR community-dwelling elderly OR elderly OR aged AND social engagement OR social activity OR volunteer activity OR volunteering OR social participation OR civic participation OR civic engagement. All fields were searched and the searches yielded 136 articles. Four papers dealing with elements of self-efficacy relevant to social participation were also included [17, 21, 30, 31]. The criteria for selecting potential items for the SOSA were as follows: (1) relevant to self-efficacy in the context of social participation; (2) clear, logical, and easily understandable for older adults; (3) relates to cognition or action. Finally, 32 items satisfying these criteria were identified.

The 32 items were reviewed by four expert academic researchers and four independent older adults who did not require long-term care insurance. The researchers specialized in public and community health nursing, and also had experience in the development of scales for independent older adults. The independent older adults were volunteers residing in Hokkaido. The content validity, face validity, and practical utility of the 32 items were assessed, and the wording and necessity of each item were evaluated according to the opinions of the researchers and older adults. Through this process, the number of items was reduced from 32 to 28. The items were scored on a four-point Likert scale (0, Not confident at all; 1, Somewhat unconfident; 2, Somewhat confident; 3, Completely confident).

### Phase 2: instrument validation

#### Participants and setting

To validate the instrument, we aimed to recruit approximately 5,000 independent, community-dwelling older adults (aged 65–75 years). To achieve this, all 5,351 community general support centers nationwide in Japan were contacted and asked to provide the questionnaire to one randomly selected person who met the inclusion criteria for the study, which were as follows: (1) aged ≥ 65 years; (2) living in the community (i.e., not in a hospital or residential care facility); and (3) living as an independent older adult, i.e., no requirement for long-term care or support. These criteria were in accordance with the Certified Level of Need for Long-Term Care National Insurance of Japan (*Kaigo Hoken* in Japanese).

Data were collected between July and August 2022 via anonymous self-administered paper questionnaires. Completed questionnaires were returned to the academic office by the older adults. A total of 1,336 participants completed the questionnaire, and 1,292 (96.7%) were included in the final analysis after excluding thawed aged < 65 years.

#### Measures

The demographic characteristics of interest included age, sex, residential status, number of years spent residing in the area, education level, employment status, living situation, and disease treatment status (Table 1). Two measures were used to assess the construct validity of the SOSA: the General Self-Efficacy Scale (GSES) [32], which classifies perceived self-efficacy as high or low (higher scores indicate greater self-efficacy), and a single question about perceived health answered on a four-point scale (1, Very healthy; 2, Quite healthy; 3, Not very healthy; 4, Not at all healthy). These scores were reversed using IBM SPSS Statistics (ver. 26.0; IBM Corp., Armonk, NY, USA), such that higher scores indicate better health.

**Table 1:** Demographic characteristics of the participants (*N* = 1,292)

		Number (%) or mean ± SD
Age (years)	Mean ± SD	72.6 ± 5.7
	Missing data	24 (1.9)
Sex	Male	566 (43.8)
	Female	719 (55.7)
	Missing data	7 (0.5)
Living status	Living alone	296 (22.9)
	Living with spouse	501 (38.8)
	Living with spouse and children	167 (12.9)
	Living with children and grandchild	151 (11.7)
	Other	167 (12.9)
	Missing data	10 (0.8)
Years spent living in the area	01–19	228 (17.7)
	20–29	152 (11.8)
	30–39	185 (14.3)
	40–49	268 (20.6)
	≥ 50	342 (26.5)
	Missing data	117 (9.1)
Education level	Elementary school/junior high school	167 (12.9)
	High school	614 (47.6)
	Vocational school	243 (18.8)
	University	235 (18.2)
	Graduate school	13 (1.0)
	Other	13 (1.0)
	Missing data	7 (0.5)
Currently employed	Yes (full- or part-time)	510 (39.5)
	No	773(59.8)
	Missing data	9 (0.7)
Living situation	I’m not in trouble	512 (39.6)
	I’m not in much trouble	538 (41.7)
	I’m in a little trouble	199 (15.4)
	I’m in trouble	39 (3.0)
	Missing data	4 (0.3)
Receiving treatment for a disease/condition	Yes	1067 (82.6)
	No	225(17.4)
Type of disease/condition	High blood pressure	553 (51.8)
	Diabetes mellitus	177 (16.6)
	Musculoskeletal diseases	157 (14.7)
	Visual impairment	152 (14.2)
	Heart disease	114 (10.7)
	Urinary system disease	113 (10.6)
	Cerebrovascular disease	65 (6.1)
	Cancer	54 (5.1)
	Respiratory disease	54 (5.1)
	Hearing impairment	51 (4.8)
	Mental disease	30 (2.8)
	Cognitive impairment	13 (1.2)
	Other	259 (24.3)

*SD* standard deviation

#### Statistical analyses

IBM SPSS Statistics (ver. 26.0) and Amos software (ver. 26.0; SPSS Inc., Chicago, IL, USA) were used for all analyses. Item analysis was used to investigate the reliability of the SOSA, and the factor structure was investigated by exploratory factor analysis. Items were excluded if the proportion of “Completely confident” responses (“pass rate”) was ≥ 80%, or if the non-response rate (“item difficulty”) was ≥ 5%, there was evidence of multicollinearity (correlation coefficients with other items > 0.7), the correlation coefficient for the “item–total analysis” was ≤ 0.6 (or *p* > 0.05), or there was no significant difference between the highest- and lowest-scoring groups in the “good–poor analysis”.

Items remaining after the item analysis were subjected to exploratory generalized least squares factor analysis (with promax rotation) [33]. According to the eigenvalues and scree plots, a four-factor solution was obtained. We then repeated the factor analysis after excluding items with loadings < 0.4, and assuming a four-factor structure. Factors with a Cronbach’s alpha ≥ 0.7 were considered reliable and construct validity was verified by confirmatory factor analysis. We examined model fit using the goodness-of-fit index (GFI), adjusted GFI (AGFI), comparative fit index (CFI), and root mean square error of approximation (RMSEA). The model was accepted if the GFI and AGFI were ≥ 0.90, and the CFI was ≥ 0.95. When the RMSEA is < 0.05 the model fit is considered good, while values > 0.1 are undesirable. We also examined criterion-related validity by correlating the total SOSA score with those of the GSES and the subjective health status scores.

## Results

### Demographic characteristics

Table 1 shows the demographic characteristics of the participants. The mean age was 72.6 years (standard deviation = 5.7). In total, 55.7% of the participants were female, 63.4% lived with their family (spouse, spouse and children, or children and grandchildren), 47.1% had resided in the same area for > 40 years, 47.6% had a high school level of education, 39.5% were employed (full- or part-time employment), 81.3% indicated that they were not “in trouble” with respect to their living situation, and 82.6% had a medical condition for which they were currently being treated.

### Item analysis

As shown in Table 2, following the item analysis of the initial 28-item version of the SOSA, none of the items were excluded on the basis of the criteria delineated above, except for four that exhibited multicollinearity (items 12, 13, 25, and 26). First, we compared items 12 and 13, and items 25 and 26, and decided to retain items 12 and 25 given their importance to the scale. Items 13 and 26 were excluded such that 26 items were retained for the factor analysis.

**Table 2:** Results of item analysis of the Self-efficacy for Social Participation scale (SOSA)

No	Item	Mean ± SD	Item difficulty^a^	Multicollinearity^b^	Item–total^c^	Good–poor analysis^d^	Pass rate^e^
1	I am able to get in touch with friends and acquaintances	2.5 ± 0.7	0.7	-	.650**	.000***	61.5
2	I am able to exchange greetings with my neighbors	2.7 ± 0.6	0.5	-	.602**	.000***	69.8
3	I am able to consult others when I have concerns	2.1 ± 0.8	1.1	-	.545**	.000***	34.8
4	I am able to make new friends and maintain relationships	1.9 ± 0.8	0.7	-	.706**	.000***	27.1
5	I am able to provide counsel for those who are in need	2.1 ± 0.8	0.7	-	.718**	.000***	31.3
6	I am able to make time to spend with friends and acquaintances	2.2 ± 0.8	0.9	-	.707**	.000***	42.0
7	I am able to take on roles when asked to do so	2.1 ± 0.8	0.9	-	.725**	.000***	32.1
8	I am able to find enjoyment in my relationships with others	2.1 ± 0.8	0.9	-	.728**	.000**	35.8
9	I am able to help each other when there is a need	2.2 ± 0.8	0.7	-	.748**	.000***	40.0
10	I am able to find enjoyment in everyday life	2.3 ± 0.7	0.6	-	.742**	.000***	40.4
11	I am able to maintain a positive attitude about getting older	1.6 ± 0.8	0.7	-	.642**	.000***	14.9
12	I am able to have the motivation to learn or start something new	1.9 ± 0.9	0.9	+	.714**	.000***	26.0
13	I am able to identify things on my own that motivate me to work hard	2.1 ± 0.7	0.9	+	.738**	.000***	29.2
14	I am able to maintain an attachment to the community in which I live	2.2 ± 0.8	0.8	-	.590**	.000***	37.0
15	I am able to positively approach everything	1.9 ± 0.7	1.4	-	.735**	.000***	21.6
16	I am able to alleviate anxiety and frustration in my own way	1.9 ± 0.7	1.0	-	.636**	.000***	18.2
17	I am able to have an interest in current affairs	2.1 ± 0.7	1.2	-	.610**	.000***	30.8
18	I am able to use the Internet to gather information necessary for my life	1.4 ± 1.1	1.0	-	.473**	.000***	18.6
19	I am able to obtain information about activities going on in the community	1.9 ± 0.9	1.0	-	.695**	.000***	24.1
20	I am able to create new activities with people in the community	1.5 ± 0.9	1.2	-	.669**	.000***	14.4
21	I am able to travel on public transport	2.2 ± 0.9	1.2	-	.535**	.000***	43.4
22	I am able to arrange my own means of transportation	2.4 ± 0.8	1.0	-	.601**	.000***	55.5
23	I am able to shop for daily necessities by myself	2.6 ± 0.7	0.9	-	.600**	.000***	65.2
24	I am able to make my own judgments about the reliability of information related to daily life	2.3 ± 0.7	1.1	-	.683**	.000***	41.7
25	I am able to manage my daily routine by myself	2.5 ± 0.6	1.2	+	.648**	.000***	54.4
26	I am able to take action after planning an activity	2.4 ± 0.7	1.0	+	.696**	.000***	48.2
27	I am able to live well while dealing with my physical condition	2.3 ± 0.7	1.2	-	.631**	.000***	38.0
28	I am able to adopt appropriate infection prevention behaviors when going out	2.4 ± 0.6	1.0	-	.639**	.000***	49.7

*SD* standard deviation

^**^P < 0.01; ***P < 0.001

^a^Non-response rate ≥ 5%

^b^Correlation coefficients between items > 0.7

^c^Correlation coefficient between the score for a given item and that of all the items ≤ 0.6 (or *p* > 0.05)

^d^Non-significant difference in mean score between the highest- and lowest-scoring groups (*p* ≥ 0.05)

^e^Proportion of “Completely confident” responses ≥ 80%

### Factor structure

The results of the exploratory generalized least squares factor analysis of the 26-item scale are shown in Table 3. The eigenvalues for the one- two, three-, four- and five-factor solutions were 12.242, 1.863, 1.368, 1.181, and 0.795, respectively. We repeated the exploratory factor analysis with promax rotation until the item factor loadings exceeded 0.4; items 2, 4, 5, 7–11, 14–18 and 28 were excluded because their factor loadings did not exceed 0.4 in any analysis. After excluding those items, differences in factor loadings between factors became apparent. Excluding items with loadings of < 0.4 yielded a four-factor solution. In the final version of the scale, 12 items were loaded onto the four factors. Factor 1 included three items (items 21, 22 and 23) and was labeled “instrumental self-efficacy” (self-efficacy for independent living and mobility). Factor 2 included three items (items 24, 25 and 27) and was labeled “managerial self-efficacy” (self-efficacy for independent management of one’s life). Factor 3 included three items (items 1, 3 and 6) and was labeled “interpersonal self-efficacy” (self-efficacy for socializing and interacting with others). Factor 4 included three items (items 12, 19 and 20) and was labeled “cultural self-efficacy” (self-efficacy for forming new habits and engaging in socially oriented behaviors). Together, the four factors explained 63.21% of the variance in SOSA scores.

**Table 3:** Exploratory generalized least squares factor analysis (with promax rotation) of the Self-efficacy for Social Participation scale (SOSA)

*n* = 1,257						
No	Item	Factor I	Factor II	Factor III	Factor IV	Total scale communality
		**Instrumental self-efficacy**	**Managerial self-efficacy**	**Interpersonal self-efficacy**	**Cultural self-efficacy**	
**22**	**I am able to arrange my own means of transportation**	**0.93**	-0.01	0.01	-0.06	0.81
**21**	**I am able to travel on public transport**	**0.79**	-0.13	-0.05	0.15	0.60
**23**	**I am able to shop for daily necessities by myself**	**0.65**	0.26	0.03	-0.10	0.68
**25**	**I am able to manage my daily routine by myself**	-0.01	**0.86**	0.00	-0.05	0.68
**27**	**I am able to live well while dealing with my physical condition**	-0.08	**0.75**	0.04	0.03	0.56
**24**	**I am able to make my own judgments about the reliability of information related to daily life**	0.12	**0.65**	-0.04	0.12	0.62
**6**	**I am able to make time to spend with friends and acquaintances**	-0.02	-0.06	**0.80**	0.13	0.70
**3**	**I am able to consult others when I have concerns**	-0.07	0.03	**0.76**	-0.07	0.51
**1**	**I am able to get in touch with friends and acquaintances**	0.10	0.05	**0.69**	-0.04	0.58
**20**	**I am able to create new activities with people in the community**	-0.02	-0.07	-0.05	**0.99**	0.85
**19**	**I am able to obtain information about activities going on in the community**	0.01	0.12	0.02	**0.68**	0.61
**12**	**I am able to have the motivation to learn or start something new**	0.10	0.11	0.16	**0.43**	0.49
Variance explained (%)		45.38	52.78	59.25	63.21	
Cronbach's alpha		0.84	0.81	0.80	0.81	0.90
Correlation coefficients (r)	Factor I	1.00	0.70	0.54	0.55	
	Factor II	0.70	1.00	0.65	0.56	
	Factor III	0.54	0.65	1.00	0.61	
	Factor IV	0.55	0.56	0.61	1.00	

### Internal consistency and validity

**Fig. 1:**
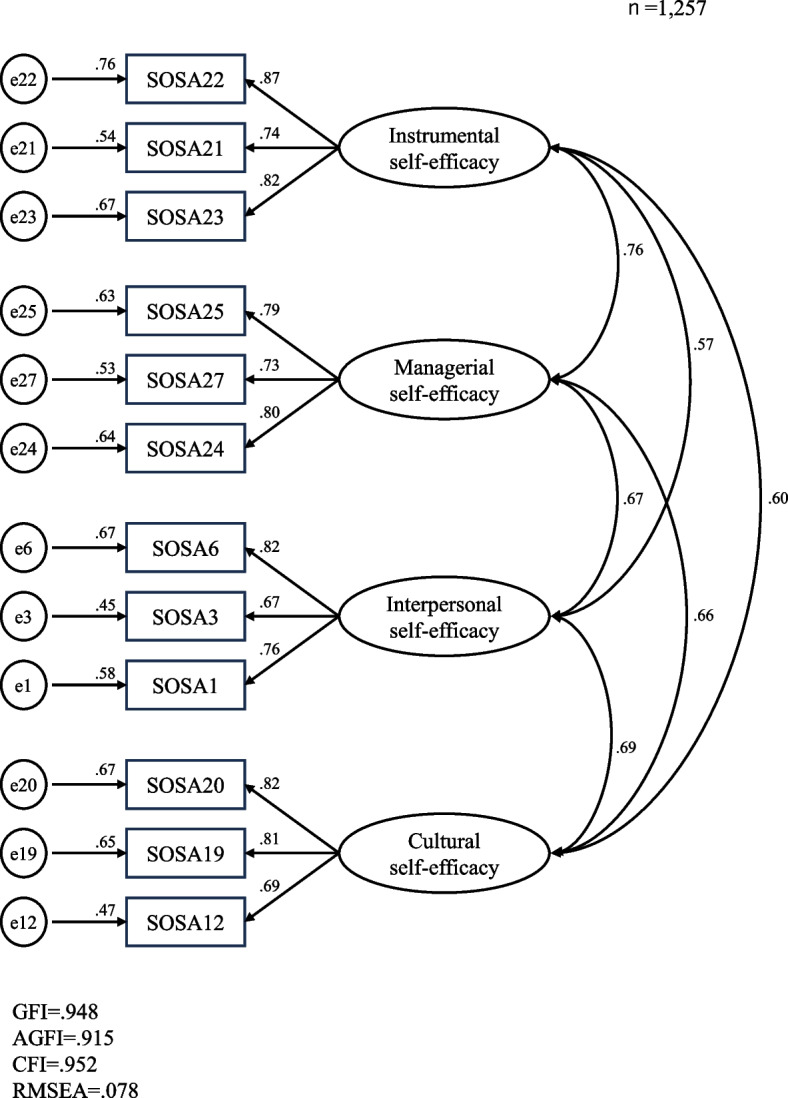
Confirmatory factor analysis of the Self-efficacy for Social Participation scale (SOSA)

The fit indices for the four-factor model were as follows: GFI = 0.948, AGFI = 0.915, CFI = 0.952, and RMSEA = 0.078 (Fig. 1). The values indicated good construct validity for the SOSA. The Cronbach’s alpha coefficients were 0.84, 0.81, 0.80, 0.81 and 0.90 for factors 1–4 and the entire scale, respectively (Table 3).

Pearson’s correlation analysis showed a correlation between the total SOSA score and GSES and subjective health status scores. The SOSA score showed a highly positive correlation with the GSES score (*r* = 0.550, *p* < 0.01) and a moderate positive correlation with the subjective health status score (*r* = 0.384, *p* < 0.01) (Table 4).

**Table 4:** Criterion-related validity of the Self-efficacy for Social Participation scale (SOSA)

*n* = 1,257			
Factors	Mean (SD)	GSES	Subjective health status
I: Instrumental self-efficacy	7.2 ± 2.1	.331**	.289**
II: Managerial self-efficacy	7.0 ± 1.7	.458**	.330**
III: Interpersonal self-efficacy	6.9 ± 1.9	.441**	.269**
IV: Cultural self-efficacy	5.2 ± 2.2	.559**	.352**
All factors	26.4 ± 6.4	.550**	.384**

Values in the “GSES” and “Subjective health status” columns are Pearson correlation coefficients between the total SOSA scores and those for the GSES and subjective health status, respectively

*SD* standard deviation, *GSES* General Self-Efficacy Scale

^**^P < 0.01

## Discussion

We developed the SOSA to evaluate the self-efficacy of community-dwelling older adults for participation in social activities, and examined its reliability and validity. The final scale comprised 12 items and four factors: instrumental self-efficacy, managerial self-efficacy, interpersonal self-efficacy, and cultural self-efficacy. The Cronbach's alpha coefficient for the scale indicated satisfactory internal consistency; this was also the case for criterion-related validity according to the correlations between the SOSA score and those for the GSES and subjective health status. Exploratory and confirmatory factor analyses confirmed the construct validity of the SOSA, which has sufficient reliability and validity to measure self-efficacy for participation in social activities among community-dwelling older adults.

The first factor of the SOSA, instrumental self-efficacy, represents older adults' self-efficacy for independent living and mobility (e.g., arranging means of transportation, using public transport, and shopping for daily necessities). Lawton distinguished seven levels of human abilities, ranging from basic life maintenance to advanced and complex social abilities [34]. The items of the SOSA relate to instrumental self-maintenance in a manner that represents the various stages proposed by Lawton. Factor 1 relates to basic self-efficacy for social participation. In a previous study, a decline in the ability to perform instrumental activities of daily living (according to a subscale of the Tokyo Metropolitan Institute of Gerontology Index of Competence) was negatively related to social participation among older adults [35]. Other studies have shown that neighborhood walkability [36] and the use of public transportation [14] promote social participation among older adults. Thus, higher instrumental self-efficacy may promote social participation by providing the means to engage in basic outings and interactions.

The second factor of the SOSA, managerial self-efficacy, represents self-efficacy for independent management of one’s life (e.g., management of the daily routine, self-care, and information). After retirement, older adults have to consciously reconstruct many parts of their lives that were previously managed by their employer, which requires the setting of new goals consistent with their values and interests, and management of their lives, while also adapting to the health status changes associated with aging [37]; these health status changes are directly associated with social participation [38]. In addition, in today’s society, the use of personal computers, cell phones, and smartphones has become widespread, and the number of older people who own such devices is increasing [39]. Information retrieval from the Internet is becoming more widespread among older adults [40]. Therefore, managerial self-efficacy as it pertains to the appropriate application of information technology to daily life is also essential for older adults to participate in society.

The third factor of the SOSA, interpersonal self-efficacy, represents self-efficacy for socializing and interacting with others (e.g., making time to spend with friends and acquaintances, consulting with others, and contacting friends and acquaintances). Previous qualitative research has identified factors that promote social participation in older adults, such as staying active (i.e., “establishing active interpersonal and social communication”) [41]. In another study, older adults who actively interacted with their neighbors were more likely to participate in social activities [21]. Other factors related to social participation and volunteer activities include good relationships with other people in the community, reflected in having a large number of close friends and receiving invitations to participate in activities [42]. In summary, interpersonal self-efficacy may promote social participation by expanding one's interests and horizons as they relate to other people.

The fourth factor of the SOSA, cultural self-efficacy, represents self-efficacy for forming new habits and engaging in socially oriented behaviors (e.g., motivation to create new activities, obtain information, and try new things). Culture has been described as “all of the behavioral patterns, arts, beliefs, customs, ways of life, and other products of a group of people that are handed down in society and guide worldviews and decision-making” [43]. Aoo et al. argued that, in Japan, older adults are often viewed as “beneficiaries” or people in need of support [44]. They also stated that, in many countries experiencing a rapid population decline, including Japan, older adults support those in need, rather than being exclusively the beneficiaries of support, and there is a need to cultivate the view that at least some older people can play a more active and responsible role in society. In a study of the requirements for social participation among older adults [15], the availability of information relevant to activities was the most important factor for engaging in a new social activity, although willingness to learn was also important [17]. Greater cultural self-efficacy may lead to a new culture of proactive social participation among older adults.

A novel aspect of the SOSA is its focus on self-efficacy for social participation. Previous measures of social participation among older adults assessed the frequency of social participation [45], motivation to engage in activities [46], attitudes toward activities [47], and confidence and beliefs relating to participation in a particular activity [27]. The SOSA is a broad measure of social participation that assesses the role of instrumental, managerial, interpersonal, and cultural self-efficacy. There are three main potential applications of the SOSA. First, it can be used to evaluate the self-efficacy of older adults for social participation and inform interventions designed for this population. Second, it can identify older adults that provide, rather than merely benefit from, social support. Third, it could lead to the creation of new community systems and policies fostering social participation among older adults, especially through comparison of a given community with others.

This study had some limitations. First, because it used a cross-sectional design, the predictive validity of the SOSA remains to be established. In the future, the longitudinal association between SOSA and social participation should be examined. Specifically, it is necessary to determine whether older people with higher SOSA scores can promote their behavioralization of social participation. Second, the response rate was moderate in this study (*n* = 1,336/5,351). Third, although questionnaires were distributed to community general support centers nationwide, regional bias in the respondents cannot be ruled out. For example, low response rates can significantly affect the generalizability of study findings. When responses are limited to specific individuals or regions, questions arise about whether the results can be accurately applied to the entire population. In such cases, the skewed representation of respondents can potentially distort results or overly reflect certain trends. Moreover, the geographical bias among respondents is an important factor. Different socioeconomic, cultural, and environmental factors exist across regions both within and outside Japan, which can influence SOSA and social participation. There are several approaches to addressing these limitations. First and foremost, it’s crucial to devise survey methods that enhance response rates by making surveys accessible to the target population in older people-friendly formats. Furthermore, to mitigate regional bias, securing an adequate sample from various regions and considering methods to appropriately adjust for regional differences both within and outside Japan are essential.

## Conclusion

The SOSA consists of instrumental self-efficacy, managerial self-efficacy, interpersonal self-efficacy and cultural self-efficacy showed sufficient reliability and validity to assess self-efficacy for social participation among older adults. This scale has the potential to help create interventions and systems, policies that could promote social participation by assessing the self-efficacy of community-dwelling older adults for social participation. Furthermore, the scale could help in efforts to improve the physical and mental health and longevity of older adults through increased social participation.

### Supplementary Information

**Additional file 1. ** The Self-efficacy for Social Participation scale (SOSA) English Version.

**Additional file 2. ** The Self-efficacy for Social Participation scale (SOSA) Japanese Version.

## Data Availability

The datasets used or analyzed during the current study are available from the corresponding author on reasonable request.

## Abbreviations

AGFI: Adjusted goodness-of-fit index
CFI: Comparative fit index
GFI: Goodness-of-fit index
GSES: General Self-Efficacy Scale
RMSEA: Root mean square error of approximation
SOSA: Self-efficacy for Social Participation scale
