# Supplementary material for: Development of the Self-efficacy for Social Participation scale (SOSA) for community-dwelling older adults
Source: BMC Public Health. 2023 Nov 20;23:2294. doi: 10.1186/s12889-023-16774-6 (PMC10662651; doi:10.1186/s12889-023-16774-6)
Supplement: Supplementary file 1 — Additional file 1. The Self-efficacy for Social Participation scale (SOSA) English Version. [file 12889_2023_16774_MOESM1_ESM.docx]

Additional file 1. The Self-efficacy for Social Participation scale (SOSA) English version

Please choose (○) the rating closest to your thought for each statement.

| No. | domain / item | Not confident  at all | Somewhat unconfident | Somewhat confident | Completely confident |
| --- | --- | --- | --- | --- | --- |
| Instrumental self-efficacy | | | | | |
| 1 | I am able to arrange my own means of transportation | ０ | １ | ２ | ３ |
| 2 | I am able to travel on public transport | ０ | １ | ２ | ３ |
| 3 | I am able to shop for daily necessities by myself | ０ | １ | ２ | ３ |
| Managerial self-efficacy | | | | | |
| 4 | I am able to manage my daily routine by myself | ０ | １ | ２ | ３ |
| 5 | I am able to live well while dealing with my physical condition | ０ | １ | ２ | ３ |
| 6 | I am able to make my own judgments about the reliability of information related to daily life | ０ | １ | ２ | ３ |
| Interpersonal self-efficacy | | | | | |
| 7 | I am able to make time to spend with friends and acquaintances | ０ | １ | ２ | ３ |
| 8 | I am able to consult others when I have concerns | ０ | １ | ２ | ３ |
| 9 | I am able to get in touch with friends and acquaintances | ０ | １ | ２ | ３ |
| Cultural self-efficacy | | | | | |
| 10 | I am able to create new activities with people in the community | ０ | １ | ２ | ３ |
| 11 | I am able to obtain information about activities going on in the community | ０ | １ | ２ | ３ |
| 12 | I am able to have the motivation to learn or start something new | ０ | １ | ２ | ３ |
| No.1-12　Total　　　　　　　　point | | | | | |

OE Nanami, TADAKA Etsuko. Development of the Self-efficacy for Social Participation scale (SOSA) for community-dwelling older adults.
