# Supplementary figures and images for: Development of the Self-efficacy for Social Participation scale (SOSA) for community-dwelling older adults
Source: BMC Public Health. 2023 Nov 20;23:2294. doi: 10.1186/s12889-023-16774-6 (PMC10662651; doi:10.1186/s12889-023-16774-6)

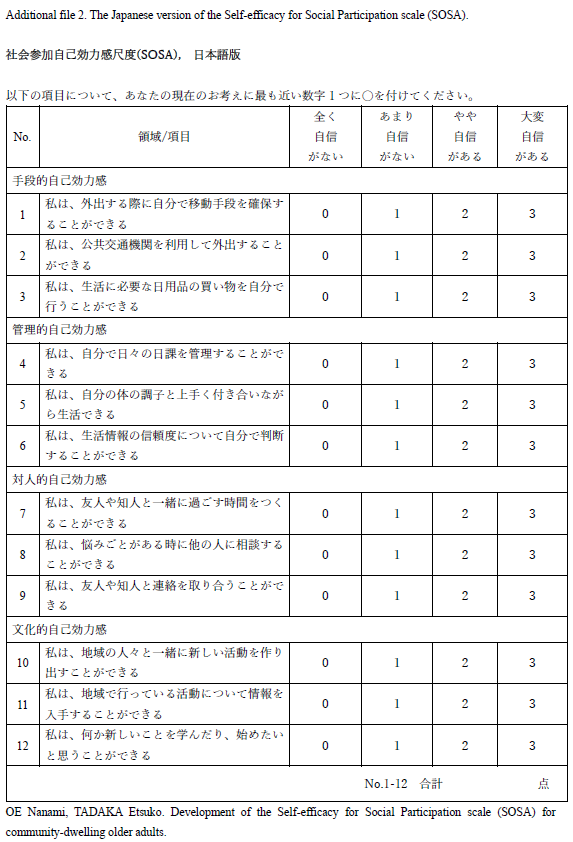

Supplement: Supplementary file 2 — Additional file 2. The Self-efficacy for Social Participation scale (SOSA) Japanese Version. [file 12889_2023_16774_MOESM2_ESM.docx]
